# Epigenetic alterations in the suprachiasmatic nucleus and hippocampus contribute to age-related cognitive decline

**DOI:** 10.18632/oncotarget.4036

**Published:** 2015-05-08

**Authors:** Scott H. Deibel, Erin L. Zelinski, Robin J. Keeley, Olga Kovalchuk, Robert J. McDonald

**Affiliations:** ^1^ Canadian Centre for Behavioural Neuroscience, Department of Neuroscience, University of Lethbridge, Lethbridge, AB, Canada; ^2^ Department of Biological Sciences, University of Lethbridge, Lethbridge, AB, Canada

**Keywords:** aging, brain, memory, circadian rhythms, Gerotarget

## Abstract

Circadian rhythm dysfunction and cognitive decline, specifically memory loss, frequently accompany natural aging. Circadian rhythms and memory are intertwined, as circadian rhythms influence memory formation and recall in young and old rodents. Although, the precise relationship between circadian rhythms and memory is still largely unknown, it is hypothesized that circadian rhythm disruption, which occurs during aging, contributes to age-associated cognitive decline, specifically memory loss. While there are a variety of mechanisms that could mediate this effect, changes in the epigenome that occur during aging has been proposed as a potential candidate. Interestingly, epigenetic mechanisms, such as DNA methylation and sirtuin1 (SIRT1) are necessary for both circadian rhythms and memory. During aging, similar alterations of epigenetic mechanisms occur in the suprachiasmatic nucleus (SCN) and hippocampus, which are necessary for circadian rhythm generation and memory, respectively. Recently, circadian rhythms have been linked to epigenetic function in the hippocampus, as some of these epigenetic mechanisms oscillate in the hippocampus and are disrupted by clock gene deletion. The current paper will review how circadian rhythms and memory change with age, and will suggest how epigenetic changes in these processes might contribute to age-related cognitive decline.

## INTRODUCTION

“I have lost my rhythm. I can't eat. I can't sleep.”

Charles Bukowski, Metamorphosis.

As suggested by the quote, factors that affect physiology, such as aging, disease, or work schedule, can affect circadian rhythms, and disruption of these daily rhythms can be debilitating. Understanding the effects of the desynchronization of daily rhythms on health and well-being is of paramount importance because a myriad of disease states have been associated with circadian rhythm disruption, including heart disease and cancer [1, 2]. Circadian rhythms and memory are among the physiological processes that decline during aging (for reviews see, [3, 4]). Memory and circadian rhythms are not separate processes, as circadian rhythm disruption can elicit memory impairments (for reviews see [2, 5, 6]). Circadian rhythm dysfunction could contribute to age-associated cognitive decline, as the degree of circadian rhythm disruption has been positively correlated with mild cognitive impairment and memory impairment in aged humans and rodents, respectively [7, 8]. It is clear that alterations in circadian rhythms influence memory, but the nature and mechanism of this relationship is largely unknown.

The hippocampus is integral to the formation of memories. Its role in learning and memory formation has been widely studied and discussed throughout human and animal research. Clock genes and plasticity molecules oscillate in the hippocampus; however, it is unclear exactly how circadian rhythms affect memory acquisition, consolidation, retention, or recall [9–12]. Epigenetics are involved in circadian rhythm generation and memory formation. These epigenetic mechanisms also display age-related alterations in key brain regions that modulate circadian rhythms and memory [4, 13]. The aims of the present article are three-fold. First, we will discuss how circadian rhythms and memory are intertwined and the specific involvement of epigenetics in these processes. Second, we will demonstrate that epigenetic modifications are involved in the age-related decline in these processes. Third, we will discuss recent data suggesting that age-related cognitive decline could be due to altered epigenetic functioning in the hippocampus elicited by circadian rhythm disruption.

### Circadian rhythms

Circadian rhythms are endogenously generated cyclical behaviours or physiological processes that take approximately 24 hours per cycle. The sleep-wake cycle, locomotor activity, temperature regulation, water/food intake, metabolism, and levels of circulating hormones are all physiological measurements of circadian rhythms [14, 15]. Zeitgebers are environmental cues (such as light pulses, social activity, meals, or exercise) that synchronize an organism's endogenously generated rhythms with the environment [16]. Animals with circadian rhythms entrained to the environment have an advantage because they are able to anticipate daily events.

Though relatively small, the suprachiasmatic nucleus (SCN) is the master pacemaker in the central nervous system. It consists of a pair of bi-lateral nuclei in the anterior hypothalamus, and SCN cells exhibit rhythmic oscillations in electrical activity and gene expression of specific genes coined ‘clock genes’, such as *Clock (Clk)*, *BMAL1*, *CRY* and *PER* [17–20]. Recently, optogenetic stimulation or suppression of SCN neurons has suggested that SCN electrical activity determines the phase/period of clock gene oscillations in SCN neurons and in turn changes circadian locomotor activity [21]. Circadian rhythms are generated at the molecular level by rhythmic gene expression, which arises from an autoregulatory transcription/post-transcription/translation/post-translational feedback loop [19]. Briefly, there are positive and negative limbs of this feedback loop, as CLOCK and BMAL1 activate the transcription of specific genes, whereas CRY and PER homologues repress the transcription of specific genes [19, 20]. CLOCK:BMAL1 heterodimers increase the transcription of CRY and PER homologues, while CRY:PER heterodimers inhibit their own transcription by decreasing CLOCK and BMAL1 expression [19, 20]. The positive limb of this feedback loop is regulated by another feedback loop, in which BMAL1 transcription is activated and repressed by RORα and REV-ERBα, respectively [19, 20].

Although the SCN is a key circadian regulator, clock genes oscillate in other brain regions [11, 22] and peripheral organs [23]. The coordination of rhythms in most peripheral oscillators (the amygdala, neocortex, cerebellum, and hippocampus) depends on the SCN [24–26]. Conversely, some oscillations are maintained in other peripheral tissues (the retina, cornea, pituitary, lung tissue, liver, olfactory bulb, arcuate nucleus, dorsomedial hypothalamus, and lateral habenula) for prolonged periods of time in the absence of the SCN [22, 27]. However, cell synchrony within and between these tissues requires an intact and functioning SCN, providing further evidence that the SCN is a key circadian regulator [22, 27].

#### Circadian rhythms influence learning and memory

A long history of research has examined the role of the time of day and light-dark cycle alterations in memory formation. Certain rodents learn better at specific times of day or only retain information when training and testing occur at the same time of day, suggesting that the circadian system is involved in memory formation and recall [10, 28–32]. Rats can use circadian timers to solve time-place-learning tasks wherein the response contingency is determined by the time of day [33, 34]. Changing the light-dark cycle daily, or photoperiod shifting, disrupts memory for passive and active avoidance tasks [35–39]. However, it is unclear if this effect is transferable to other types of memory.

Episodic memories, which are memories for the time and place of personally experienced events, are particularly affected by aging [40, 41]. In humans, disease pathologies, single unit recording, and functional magnetic resonance imaging have confirmed that the hippocampus is required for the encoding and recall of episodic memories [42–44]. With this in mind, we hypothesized that circadian rhythms might disrupt hippocampal-dependent memory in rodents [45]. Male rats exposed to a consecutive six-day photoperiod shifting paradigm, wherein the lights came on three hour earlier each day, had normal acquisition in the standard, spatial version of the Morris water task (MWT), but displayed impairments in long-term retention [45]. Longer periods of photoperiod shifting (e.g., several weeks) impair acquisition in this same spatial task [46]. While these data suggest that circadian rhythm disruption interferes with memory consolidation, it was unclear if circadian disruption could interfere with previously consolidated long-term memories. Animals that had acquired the spatial version of the MWT and were then exposed to brief or lengthy periods of photoperiod shifting had various degrees of hippocampal-dependent memory impairment, despite retention testing being conducted when the animal's circadian rhythms had re-entrained [5, 6]. Additionally, photoperiod shifting alters performance in the hippocampal-dependent context-based discriminative fear conditioning tasks in mice and rats [47, 48]. Thus, circadian rhythm disruption can affect consolidation and retrieval of long-term memories.

**Table 1:** Key studies in chronological order that have demonstrated that circadian rhythm disruption elicit memory impairments in rodents

Study	Animal Sex/Strain/Species	Manipulation Used to Induce Circadian Rhythm Disruption	Behavioural Task Used to Detect Memory Impairment
38	Male albino rats	Photoperiod shifting	Passive avoidance task
7	Male golden hamsters	Natural aging	Appetitive contextual conditioning
45	Male Long-Evans rats	Acute photoperiod shifting	Spatial water task
46	Male Long-Evans rats	Acute and chronic photoperiod shifting	Spatial water task
56	Male and female Siberian hamsters	Light pulse/phase delay	Novel object recognition task
49	C57BL6/J background mice	*Cry1Cry2* mutation	Time-place learning task
50	Male C57BL/6 background mice	*Per2* mutation	Trace fear-conditioning
48	Male C57BL/6 mice	Photoperiod shifting	Contextual fear conditioning
11	Male C3H/J background mice	*Per1* mutation	Spatial radial arm maze
51	Male C57BL/6J background mice	*Bmal1* mutation, *Clk* mutation	Open field context exploration
12	Male C57BL/6 mice	SCN lesions	Contextual fear conditioning and spatial water task
5	Male and Female Long-Evans rats	Chronic photoperiod shifting	Spatial water Task and stimulus response visual discrimination task
57	Female Long-Evans rats	Chronic photoperiod shifting	Appetitive context discrimination
52	Male C57BL/6 background mice	*Bmal1* mutation	Contextual fear conditioning and spatial water task
32	Male C3H/HeN background mice	*Per1* mutation	Spatial working memory task
6	Male and Female Long-Evans rats	Acute photoperiod shifting	Spatial water Task

In addition to environmental manipulations, physiological manipulations that affect circadian rhythmicity, such as clock gene mutations (*Per1−/−*: 11; *Cry1−/− Cry2−/−*: 49; *Per2−/−:* 50; *Bmal1−/−*: 51; 52; *Clk*: 51) or SCN lesions [12,53], can also elicit hippocampal-dependent memory impairments in rodents. Further, some cognitive disorders in humans (e.g., bipolar disorder) exhibit characteristic single nucleotide polymorphisms in several canonical clock genes [54, 55].

The precise mechanisms responsible for the influence of circadian rhythms on memory are unknown. There are a variety of possible mechanisms [2, 58]. Clock genes and plasticity molecules, such as mitogen-activated protein kinase (MAPK), cyclic adenosine monophosphate (cAMP), Ca^2+^-stimulated adenylyl cyclases, and Ras, exhibit circadian oscillations in the hippocampus [10–12, 50, 59, 60]. All of these molecules have roles in long-term potentiation (LTP), the hallmark hippocampal processes thought to underlie memory formation. Thus, LTP decay and the magnitude of enhancement also oscillate [61, 62], and not surprisingly, mice with clock gene knockouts display impaired LTP (*Per1−/−*: 32; *Per2−/−*: 50; *Bmal1−/−*: 52). However, the circadian disruption experienced during shift work, transmeridian travel, and aging is not due to the deletion of clock genes, but instead it is believed to be due to an uncoupling of peripheral oscillators and the SCN [2, 63, 64]. Environmental light manipulations, such as photoperiod shifting, mimic this effect, as the phase relationship between clock proteins in the hippocampus and the SCN is changed in hamsters exposed to constant dim-illumination conditions [65]. Similarly, as suggested by Sidor and McClung [66], recently developed chronic optogenetic preparations might be useful in desynchronizing SCN oscillations from those in peripheral oscillators like the hippocampus.

Evidence to date suggests that the hippocampal oscillation of clock genes and plasticity mechanisms are driven by the SCN. For example, *Per2* oscillations in the dentate gyrus region of the hippocampus are dependent on the SCN [24]. However, *Per2* will continue to oscillate in all hippocampal sub-regions in slice preparations (though coherence degrades over time), suggesting that the hippocampus is capable of semi-autonomous oscillations [50]. Additionally, the oscillation of synaptic plasticity molecules is dependent on the SCN [12], suggesting that clock gene oscillations are contributing to the rhythmic activity of these plasticity molecules in the hippocampus. For example, the rhythmic phosphorylation of cAMP binding protein (CREB) is dependent on *Per1* expression [32]. Similarly, MAPK and cAMP rhythms in the hippocampus are dependent on *Bmal1* expression [52]. Since SCN ablation produces similar effects to global clock gene mutations, it is possible that hippocampal rhythms of plasticity molecules are dependent on hippocampal clock gene oscillations that are abolished by SCN lesions. Optogenetic manipulation of SCN activity, or hippocampus and SCN-specific clock gene knockout animal models would address this question.

There are a number of theories as to how circadian rhythms modulate memory. Memory formation in the hippocampus and zeitgeber responses in the SCN induce very similar molecular cascades [9]. In a sense, memory encoding might be time-stamped by integrating the current time of input with molecular oscillations [9]. Thus, Eckel-Mahan and Storm [9] propose that memory encoding acts as a zeitgeber in the hippocampus by synchronizing molecular oscillations or by changing the rhythmic activity of neuronal ensembles. Recent empirical data support this view, as Ralph and Colleagues [67] demonstrated that the retention of a place memory (contingent on a specific time-of-day) eventually drifts to a new time in the absence of training and that this drift is dependent on the SCN. The authors hypothesized that there is a context-entrainable oscillator (CEO) that requires persistent input (training) to maintain time-of-day memory, and in the absence of memory training, the SCN acts as a weak zeitgeber that eventually entrains the CEO [67]. The hippocampus is a likely candidate for this CEO, as the task used in this study is hippocampal-dependent, and some of the molecular mechanisms of hippocampal-dependent memory are under circadian control.

In summary, circadian rhythms are involved in learning and memory, and hippocampal clock gene dysregulation and plasticity changes in specific biochemical plasticity pathways are likely responsible for the memory impairments associated with circadian rhythm disruption. However, there are other possible mechanisms by which circadian rhythm disruption could influence clock gene activity or other memory processes. Epigenetic changes have been proposed as a mechanism for circadian rhythms' modulation of memory because they are involved in the circadian clock and memory [58].

### Epigenetics

Prior to the discovery of epigenetics, DNA was thought to determine phenotype exclusively. Phenotype can also be altered without a change in the underlying genome sequence, and this change is a result of epigenetic modifications [68]. Cell nuclei contain chromosomes that are composed of a complex of DNA and proteins called chromatin [69, 70]. The repeating basic units of chromatin - nucleosomes - contain 147 base pairs of DNA wrapped tightly around octamers of histone proteins [69–71]. Epigenetic modifications change chromatin state, producing states that either promote or suppress DNA transcription [70]. Heterochromatin consists of compacted chromatin and is associated with reduced gene expression [70, 72], whereas euchromatin promotes gene expression, as the chromatin is in an elongated state [72, 73]. The two main epigenetic modifications include histone modification or direct action on DNA, such as DNA methylation [70, 72], and these changes can be transient [74] or long lasting and heritable [72, 74].

Histone modification occurs through enzymatic activity at the histone tail [69, 70, 72] and affects transcription by either elongating or condensing chromatin [70]. Although the specific function of a histone modification often depends on the conditions at that time, they can be broadly classified as either activating or repressive [70]. Acetylation and phosphorylation primarily activate transcription, while histone methylation, sumoylation, deacetylation, deamination, and proline isomerization are primarily repressive [70]. Furthermore, ubiquitination can be either activating or repressive, and even methylation can activate transcription in certain situations [70]. Some examples of molecules involved in histone modifications are those that add (histone acetyltransferase enzyme (HAT)) or remove (histone deacteylase enzyme (HDAC) acetyl groups from histones [70].

DNA methylation is the most commonly studied epigenetic mechanism, and in contrast to histone modifications, it exclusively represses transcription [72, 75]. DNA methylation typically involves the addition of methyl groups to cytosine bases that are bound to guanine via a phosphodiester bond (CpG dinucleotides) [72, 73, 75]. DNA methyltransferase enzymes facilitate DNA methylation by adding methyl groups to DNA [75]. The areas in the genome (primarily found in promoter and first exon regions of the DNA sequence) that have a high concentration of CpG dinucleotides are referred to as CpG islands [72, 73, 75–78]. CpG islands are typically umethylated so that transcription can occur [73, 75]. Thus, the methylation of CpG islands down-regulates gene expression by blocking transcription [73]. In mammals, DNA methylation serves various functions, such as forming heterochromatin, imprinting, and X-chromosome inactivation [72]. However, variations in the amount of methylation over time can have deleterious effects. For example, in cancer cells, hypermethylation of tumor suppressing genes and hypomethylation of oncogenes can promote tumor growth and cancer progression [77, 79–85].

DNA methylated phenotypes can be transgenerationally heritable, whereas phenotypes due to histone modifications are generally considered non-heritable [86]. Unlike the relatively stable genome, the epigenome fluctuates and can change as a result of stochastic variation, aging, or exposure to environmental factors, such as nutrition and toxins [86, 87]. Epigenetic changes are more likely to occur during gestation rather than in adulthood, as chromatin modifiers are more active in early embryonic cells compared to adult cells [86]. Nonetheless, epigenetic changes also occur in adults [73, 86]. In adults, epigenetic changes are not global, and some tissues are more susceptible than others to specific environmental factors and their influence on epigenetic modifiers [86]. For example, chronic sun exposure and smoking preferentially elicit DNA methylation changes in skin cells and lung tumor suppressor genes, respectively [88, 89]. Despite the negative connotations of epigenetic alterations, epigenetic changes can be beneficial, as they allow an organism to adapt to a fluctuating environment [74].

#### Epigenetics and the circadian clock

Epigenetic modifications show circadian rhythmicity and are regulated by core-clock genes [32, 90]. In the SCN, DNA methyltransferase expression and phosphorylation of H3 histone tails are affected by the light-dark cycle and light pulses, respectively [91, 92]. Histone modifications can also be expressed rhythmically, as the acetylation of histones in *Per1*, *Per2*, and *Cry1* promoter regions has been shown to display circadian variation in mouse livers [93]. There is considerable variability in oscillatory expression of genes between tissues [94–96]. Consequently, Masri and Sassone-Corsi [97] suggested that peripheral tissue-specific gene oscillations might be a result of circadian fluctuations in chromatin states, which interact with the core clock machinery.

Since epigenetic mechanisms display rhythmic activity, not surprisingly, epigenetic modifications are directly involved in the core clock gene architecture. In addition to being a transcription factor, CLOCK is a HAT [98, 99]. CLOCK acetylates histones and non-histone proteins, such as its transcription partner, BMAL1, in a rhythmic fashion, which suggests that epigenetic modification is crucial to circadian rhythm generation [98, 99]. Interestingly, another epigenetic mechanism modulates the activity of BMAL1:CLOCK heterodimers, as a histone methyltransferase - mixed-lineage leukemia 1 (MLL1) - enhances the transcriptional activation properties of BMAL1:CLOCK heterodimers and is required for the oscillation of all the core clock genes [100].

Given that CLOCK activates transcription by remodeling chromatin, researchers have hypothesized that there might be a circadian clock component that epigenetically represses transcription. The HDAC, sirtuin1 (SIRT1) is a likely candidate [101, 102]. SIRT1, which is highly expressed in the hippocampus, cortex, cerebellum, and hypothalamus [103], is a metabolic sensor that affects cellular energy via its cofactor and activity regulator, nicotinamide adenine dinucleotide (NAD^+^; [13, 102]). SIRT1 is involved in a variety of functions, such as neurogenesis, synaptic plasticity, DNA repair, cell cycle arrest, cell survival, gluconeogenesis, lipid metabolism, insulin sensitivity, and protection against brain pathology associated with AD [13, 102, 104, 105].

SIRT1 was recently discovered to have a role in the molecular generation of circadian rhythms. The central function of SIRT1 is likely epigenetic [106], and SIRT1 contributes to circadian rhythmicity by rhythmically deacteylating BMAL1 and PER2 [107, 108]. SIRT1 primarily down-regulates BMAL1:CLOCK initiated transcription by affecting CLOCK and BMAL1 acetylation [107, 108]. SIRT1 oscillates in the SCN and hippocampus [32, 109]. There is a regulatory loop in the SCN consisting of the oscillatory proteins SIRT1, PPARg coactivator 1a (PGC-1a), and the NAD+ synthetic enzyme nicotinamide phosphoribosyltransferase (NAMPT; [109]). Together, this regulatory loop influences aspects of circadian rhythmicity, such as the free-running period [109]. SIRT1 directly regulates circadian rhythmicity, as mice lacking SIRT1 in brain [109] or liver tissue [108] had longer free-running periods/reduced activity and disrupted clock gene expression in the liver, respectively. Conversely, mice over-expressing SIRT1 in the brain had shorter free-running periods and increased amounts of activity [109].

#### Circadian rhythms and epigenetics interact synergistically

Chronotype refers to an individual's preferred time of day (morning or evening) and sleep schedule [110, 111]. Genetics and zeitgeber exposure are thought to determine chronotype [110, 111]. Barclay and colleagues [111] demonstrated that monozygotic twins exposed to different life experiences had different chronotypes, indicating that environment can influence chronotype. A study in mice indicated that chronotype can vary within inbred mice that have very similar DNA [112]. Interestingly, these changes arose in mice that were housed in identical environmental conditions. However, the authors suggest that endogenous epigenetic changes could have influenced chronotype, and this fits with findings suggesting that the epigenome can vary stochastically, independent of environmental manipulations [86, 112].

Although these studies in animal models suggest that epigenetic mechanisms influence chronotype, this phenomenon has not received much attention in the literature on humans. Only a handful of studies have suggested that DNA methylation influences chronotype in humans. For example, the gene for the cytokine tumor necrosis factor alpha (TNF-α), which among other things is involved in cell survival, had higher levels of methylation from peripheral blood samples collected from shift workers with a late chronotype rather than an early one [113]. Similarly, there was a nonsignificant trend for late chronotypes to have increased CLOCK gene methylation expression in white blood cells [114].

Similar to the notion that epigenetics can affect chronotype or circadian phenotype, epigenetic modifications are involved in entrainment to a new photoperiod. In this study, there were global transcriptome changes in DNA methylation in the SCN of mice that had entrained to a novel photoperiod [91]. The authors hypothesized that free-running period adjustment is mediated by DNA methylation. This conclusion seems likely, as a DNA methyltransferase inhibitor, which decreases DNA methylation, prevented entrainment to the novel photoperiod [91]. Shorter photoperiods reduce DNA methylation of a hypothalamic gene involved in sexual reproduction, suggesting that reversible epigenetic modifications are involved in adapting physiology to seasonal environmental variations [115]. Collectively, these data suggest that epigenetic changes induced by environmental manipulations or internal stochastic variation can influence the free-running period of circadian rhythms.

#### Circadian rhythm disruption influences the epigenome

Since epigenetic modifications are involved in circadian rhythms, it is likely that epigenetic variation elicited by circadian rhythm disruption can negatively impact health. It is believed that shift-workers are prone to various disease states because they are in a state of constant circadian disruption [116–118]. Changes in the epigenome have been suggested as a possible mechanism for the deleterious effects of circadian disruption on health [58, 73, 119].

Various studies have investigated DNA methylation in shift workers. One study demonstrated that shift-workers have reduced CLOCK and increased CRY2 methylation in peripheral blood samples relative to day workers [119]. The association between altered metabolic processes and shiftwork is exemplified via the link between shiftwork and cancer [120]. Breast cancer rates are elevated amongst shift-workers, and breast cancer patients express CLOCK hypomethylation [121] and CRY2 hypermethylation [122] in blood and tumorous breast tissue. In a phenomenon called genomic imprinting, epigenetics can silence an allele inherited from one of the parents so that gene expression is determined from only one inherited allele [123]. Several imprinted genes associated with cancer were differentially methylated in blood samples from shift-workers compared to day workers [124]. The amount of time working on a shiftwork schedule also affects the epigenome, as Bolatti et al. [113] showed increased global hypomethylation on specific repetitive elements in the blood of long-term shift-workers. Interestingly, in these long-term shift-workers, TNF-α and a cytokine involved in tumor regulation, interferon-gamma (IFN-γ), were also hypomethylated [113].

Free-running periods are affected by epigenetic modifications. A micro RNA (miRNA) involved in regulating free-running circadian rhythm period length (miR-219) was hypermethylated in the blood of female shift workers [125]. These data support the notion that DNA methylation and changes in miRNA activity, which is another epigenetic mechanism, are involved in regulating the period of circadian rhythms, as these workers were likely in a state of chronic circadian disruption [116–118].

Very few animal studies have investigated the effects of circadian rhythm disruption on the epigenome. Human shift-work data have been corroborated in mice via the demonstration that light periods during the normal dark phase (aka, light at night; LAN) elicit global DNA hypomethylation in breast cancer tumors [126]. Melatonin is a rhythmically expressed hormone secreted by the pineal gland that, in addition to being a hormonal regulator of circadian rhythms, also suppresses the growth of breast cancer cells [73]. Melatonin release is attenuated and non-rhythmic in circadian disrupted individuals, which might contribute to the higher prevalence of cancer in shift workers [73, 126]. Interestingly, this effect is thought to be due to melatonin's hypothesized role in silencing genes via epigenetic mechanisms [73]. Thus, Schwimmer and colleagues [126] demonstrated that exogenous melatonin supplementation significantly reduced tumor growth and hypomethlyation that was induced by LAN, suggesting that melatonin suppression may be the mechanism underlying the consequences of LAN on DNA methylation. Similarly, in a rat model, we demonstrated that chronic circadian rhythm disruption induced by a lengthy period of photoperiod shifting, up-regulated some miRNAs that are indices of cancerous tissue and down-regulated some miRNAs involved in tumor suppression (Kovalchuk & McDonald, unpublished).

As a whole, data indicate that circadian rhythm disruption changes the epigenome, but the permanence of epigenetic changes induced by circadian disruption is unknown and requires further investigation. Similar epigenetic changes in shift workers and cancer patients support the hypothesis that shift work acts as a carcinogen. One caveat of the human shift-work studies mentioned above is that most of them assessed DNA methylation in peripheral blood [121]. Epigenetic modifications can be tissue specific [86], and, as with any environmental manipulation or toxin, it is likely that some tissues are more susceptible to the harmful effects of circadian disruption. Increasing the number of studies implementing animal models of shift work will ensure a more thorough understanding of the effects of circadian disruption on the epigenome.

#### Epigenetics regulation of memory formation and recall

Epigenetic changes, such as histone acetylation, phosphorylation, methylation, and especially DNA methylation, are involved in memory [127, 128]. Learning of hippocampal-dependent tasks induces epigenetic changes in the hippocampus and other brain areas to support memory formation. For example, the acetylation of histones in the hippocampus is increased shortly after contextual fear conditioning and MWT training [129, 130]. The nature of the behavioural task employed dictates which brain areas will undergo epigenetic changes. For example, acetylation occurs in the hippocampus and striatum during hippocampal- and striatal-based tasks, respectively, which suggests that this acetylation is associated with learning [130]. Furthermore, transgenic mice with disrupted CREB binding protein (CBP), which is a HAT, are impaired in various hippocampal-dependent tasks [131, 132]. Furthermore, the inhibition of an HDAC in a mouse model rescued fear conditioning retention and late-phase LTP [131]. In these studies, the impairments in spatial learning tasks - the gold standard measure of hippocampal functioning - were exclusive to CBP transgenic animals, in which the HAT region is specifically targeted, rather than a partial CBP deletion [128, 131, 132]. Inhibiting HDACs can enhance aspects of contextual fear conditioning and spatial memory, which further supports an integral role of histone acetylation for memory [128, 131, 133, 134]. Histone phosphorylation is also involved in hippocampal-dependent memory, as H3 phosphorylation, which is dependent on the ERK/MAPK pathway (under circadian control), is increased in the CA1 region of the hippocampus following fear conditioning [135].

Epigenetics is likely a conserved mechanism. Consequently, some of the epigenetic modifications involved in circadian rhythms (e.g., DNA methylation) are shared with learning and memory. DNA methyltransferase expression, which facilitates DNA methylation, is increased in the hippocampus following contextual fear conditioning [136]. Blocking these training-induced changes in DNA methylation shortly after [136] or 30 days [137] after training resulted in memory impairments. Training-induced changes in DNA methylation are gene dependent, as increased demethylation and methylation of memory promoting and suppressing genes, respectively, occur in the hippocampus shortly after contextual fear conditioning [128, 136]. In one study, DNA methylation was present in the anterior cingulate cortex up to 30 days after contextual fear conditioning, suggesting that learning induced epigenetic changes can be long lasting [137].

SIRT1, an HDAC involved in the autoregulatory feedback loops of circadian rhythm generation, is also necessary for long-term memory [104]. SIRT1 regulates miRNA-134, which affects the brain-derived neurotropic factor (BDNF) and CREB [104, 128]. Both BDNF and CREB are required for synaptic plasticity, which is crucial for learning and memory [4, 104, 138–141]. In addition to having disrupted circadian rhythms, SIRT1 knockout mice have impaired LTP, and these mice are impaired in contextual and cued fear conditioning, short-term memory, and spatial learning (MWT; [104, 105, 109]).

### Aging

#### Circadian rhythms degrade with age

Many aspects of circadian rhythmicity are affected by aging [3]. Changes to the length [142–147] and amplitude [144, 146–149] of the activity period have been observed in rodents. Rhythm fragmentation in rodents increases with age and results in the timing of behaviour or other processes under circadian control occurring at atypical times of day [147, 150]. Finally, the circadian clock is not as plastic with advanced age, making it harder for rodents to entrain to new light-dark cycles [91, 109, 147].

In addition to activity rhythms, aging affects other circadian processes. For example, daily fluctuations in hormone levels are disrupted in aged humans [151, 152] and other animals [28, 153]. In animal models, corticosterone and melatonin are strongly affected by aging, as basal levels can change, or the hormones no longer oscillate [28, 153–155]. In humans, the peak amplitudes of temperature and melatonin expression decrease with age; however, men may be more affected [151, 156–158].

Sleep is also affected by aging, with the amount of rapid-eye-movement sleep increasing and the amount of slow-wave sleep (SWS, a stage of non-rapid-eye-movement sleep) decreasing with age. Some theorize that episodic memory consolidation occurs during SWS, which decreases with age [159]. Other components of sleep that are hypothesized to contribute to memory consolidation, such as K-complexes and spindles, are also reduced in aged humans [160]. Similarly, a person's chronotype changes with age, as early chronotypes predominate in the elderly and not in younger or middle-aged people [110].

It is generally assumed that the impairments in circadian rhythm output are due to aging-induced changes in SCN functioning. Although SCN cell loss is not common in aged rodents, neurotransmitter modulation of SCN function is not as effective, and the subpopulations of cells expressing these molecules are often diminished in aged individuals [161]. For example, the number of GABAergic synapses [162] and VIP/VIP receptor expression are attenuated in the SCN of aged rodents [161, 163, 164]. As a result, aging SCN cells lose synchronicity, which could affect circadian rhythm amplitude and period [161]. Circadian rhythms are less plastic in aged individuals, and SCN activity in response to light is diminished in aged rodents and primates [165–167]. Several studies demonstrate that a malfunctioning SCN contributes to circadian rhythm deterioration in aged individuals; remarkably, transplanting fetal SCN tissue into the third ventricle of an aged animal partially rescues circadian rhythmicity in rats and hamsters [165, 168–170].

The oscillatory expression of some clock genes in the SCN of rodents is affected by aging; *Per1* [171]*, Per2* [171, 172]*, Cry1* [171]*, Cry2* [171]*, Clk* [173, 174], and *Bmal1* [171, 173] mRNA oscillations are disrupted or abolished in aged rodents. Oscillating tissue outside of the SCN is also affected by aging, as the amplitude and/or rhythmicity of *Clk* and *Bmal1* are altered in the hippocampus and other brain regions of aged mice [174]. Changes in the phases of *Per1, Per2,* and *Bmal1* oscillations are observed in the brain tissue of AD patients [175]. In addition to molecular oscillations, oscillatory electrical activity is also affected by aging. The peak of electrical activity in the SCN usually occurs during midday; however, in aged mice, similar to locomotion, there is more electrophysiological activity during the expected nadir [176]. Similarly, the amplitude and period of SCN neuron electrical oscillations in *in vitro* preparations is affected by aging [177].

While it is clear that circadian rhythms deteriorate with age, it appears that this relationship is synergistic because circadian rhythmicity affects longevity. Tau mutant hamsters, who display decreased life spans, possess naturally fragmented circadian rhythms, and transplanted fetal SCN tissue into the third ventricle of aged mutants increased their lifespan [168]. Similarly, Tranah and colleagues [178] observed that elderly women with fragmented circadian rhythms had shorter life spans. It is possible that clock gene expression altered by aging might be the mechanism responsible for decreased longevity in circadian-disrupted individuals, as *Bmal1* [179] and *Clk* [180] knockout mice have reduced life spans. *Bmal1* knockout mice also have age-related pathologies, such as sarcopenia, osteoporosis, cataracts, less subcutaneous fat, decreased hair growth, organ shrinkage, and changes in peripheral blood composition [179]. Alternatively, circadian rhythm disruption could affect the expression of other proteins involved in aging and longevity such as apolipoprotein E [181] and D [182] proteins, which are involved in lipid transport.

Although the SCN is likely responsible for the circadian rhythm age-related decline, the mechanisms eliciting the SCN malfunction are still largely unknown. Recently, epigenetic modifications have been proposed as an alternative mechanism for the degradation of circadian rhythms [13, 91, 109].

#### Memory declines with aging

As discussed in a thoughtful review by Penner and colleagues [4], age-related memory impairment is common and can be debilitating. The degree of memory impairment varies from the more prevalent cases of mild, age-associated memory impairment to the rarer cases of severe, profound dementia [4, 183]. Normal aging [40] and dementias, such as AD [184, 185], are characterized by progressive loss of the ability to form and recall episodic memories. Hippocampal-dependent spatial navigation tasks are commonly used as a model for episodic memory in humans and animals [186–188]. Aged rodents are impaired in a myriad of hippocampal-dependent measures, including spatial navigation tasks [4, 130, 188–197].

Hippocampal cell death in aged humans, monkeys, and rodents is minimal [198–201]. However, the aged hippocampus is more sensitive to the negative impacts of damaging agents. For example, aged rats given mini-hippocampal strokes had larger lesions, more damaged cells, and exacerbated spatial impairments relative to younger rats [202]. Despite there being little concomitant hippocampal cell death associated with aging, various aspects of hippocampal functioning are compromised in aged individuals. In rats, the induction and maintenance of LTP are affected by aging [4, 188, 189, 203–207].

The minimal hippocampal cell death associated with aging implies that it is likely that plasticity mechanisms are affected by aging [4]. Various molecular mechanisms are required for memory [208], but the immediate early gene BDNF is of particular interest [209]. BDNF is widely expressed in the hippocampus and is involved in synaptic plasticity and thus memory [4, 139]. BDNF is up-regulated after training in hippocampal-dependent memory tasks [4, 139–141], and LTP/hippocampal-dependent memory is impaired in BDNF knockout mice [4, 139, 210, 211]. Hippocampal expression of BDNF and its receptor can be decreased in middle-aged and aged rats, although this effect is strain specific [4, 212–215]. In addition to BDNF, other immediate early genes are also down-regulated in the hippocampus of aged rats with concomitant memory impairments [4, 216, 217].

Another way of assessing hippocampal function is to assess electrophysiological correlates in freely moving animals. Hippocampal place cells fire when an animal is in a particular location of the environment [218, 219]. Recently, place cells have been hypothesized to be involved in more than the representation of spatial locations [220, 221]. For example, place cells are involved in the memory for sequences of events that comprise behaviour, as certain place cells will only fire depending on where the animal has just been or where it is planning to go [220, 222–225]. Several aspects of place cell functioning in rats are affected by aging. For example, in aged rats, place cells have decreased spatial specificity, and they are less likely to change their location when cues are altered in a familiar environment [191, 226, 227]. Furthermore, during the first few minutes of exposure to a novel environment, place fields expand [228], and this experience-dependent plasticity is abolished in aged rats [197]. Place field stability is also affected by aging; Barnes et al. [191] showed that although place fields of aged rats were stable while in an environment, these cells would remap when the animals were returned to a familiar environment. Similarly, the tuning of place cells to spatial cues and place field stability are impaired in aged mice [229].

The hippocampus replays activation sequences of previously experienced behaviours while awake [230] and at rest [231–233]. While the exact function of replay is still under debate [234], some of the literature suggests that it is involved in the consolidation or retrieval of episodic memories [233]. Age-induced memory impairment might occur because memory consolidation is impaired by disrupted replay, or memory reactivation [193]. Gerrard and colleagues [194] observed that sequential reactivation was impaired in aged animals, such that the temporal bias for a cell to fire just prior to the firing of another cell was abolished. The altered neurobiology was corroborated behaviourally as animals with poor sequential reactivation displayed greater impairments in a hippocampal-dependent task than those with superior sequential reactivation. These results suggest that aging affects hippocampal replay, and the memory impairment in aged animals could be partially due to the failure of cells to be reactivated in the correct temporal order during sleep.

#### Circadian rhythm disruption might contribute to age-related cognitive decline

The link between circadian rhythms and memory is further bolstered by data from aged individuals. For example, age alone is not the best predictor of cognitive impairment, as Antoniadis et al. [7] demonstrated that older hamsters with disrupted circadian rhythms were more impaired in a hippocampal-dependent task when compared to hamsters of the same age with entrained circadian rhythms. This effect has been observed in humans, as older women with weak circadian rhythms are reportedly more likely to develop dementia or mild cognitive impairment than age- and health-matched women with robust circadian rhythms [8].

Age-associated dampening of hormonal rhythms, such as melatonin and corticosterone, contribute to age-associated memory impairments. Hamsters with decreased baseline corticosterone and corticosterone rhythm amplitude showed greater impairments in a hippocampal-dependent task than aged-matched controls with robust corticosterone rhythms [28]. Cermakian and colleagues [175] have reported reduced synchronization of clock gene activity across brain regions in Alzheimer's patients, including the pineal gland - an important site for the secretion of melatonin. Attenuated hormonal rhythms that arise during aging could affect clock gene oscillations in the SCN and peripheral oscillators, as exogenous melatonin has been shown to improve SCN clock gene rhythmicity in aged animals [171].

Furthermore, place cell stability, formation, and reactivation are compromised in aged rodents [191, 193, 194, 197, 226, 227, 229], and in rats, the firing of hippocampal CA1 pyramidal (place) cells display circadian rhythmicity that is thought to be entrained by entry into the environment or availability of food [235]. The authors hypothesized that this rhythmic activity might be involved in encoding temporal information. It is possible that circadian rhythm disruption affects place cell activity and thus could be contributing to age-associated memory impairment.

Circadian disruption impairs memory and is correlated with the degree of memory impairment in aged animals. These results suggest that circadian disruption contributes to hippocampal pathology in aged individuals.

#### The epigenome changes during aging

Epigenetics represent an interesting mechanism for age-related changes in phenotype because the epigenome is heritable but also affected by the environment [236]. The epigenome changes with age, as in humans, global DNA hypomethylation is generally associated with aging [236–239]. Not surprisingly, DNA methyltransferases (DNMT1 and DNMT3a) are reduced during aging [239]. Histone acetylation and methylation patterns also change with age [240–242]. Epigenetic changes are also associated with AD, as the amount of DNA methylation and hydroxymethylation (oxidized DNA methylation) in the brains of AD patients is positively correlated with markers of AD, such as beta amyloid, tau, and ubiquitin load [243].

Work from Kovalchuk and colleagues have contributed to the notion that epigenetic changes occur in senescent (involved in the terminal cell cycle arrest of cells) cells. For example, DNA methylation and histone methylation (K20-H4) are significantly reduced in breast cancer cells, and this reduction is likely due to the attenuated expression of molecules involved in histone and DNA methylation, such as the histone methyltranferase (SUV39H1) and methyl binding proteins [79]. Lung cancer senescent cells also demonstrate global DNA hypomethylation [244], as well as reductions in global histone trimethylation (H3K9 trimethylation) and a histone methyltransferase (SUV39H1) [245].

Epigenetic changes are often tissue specific, as environmental factors affect some tissues more than others [86]. For example, age-related decline in the immune response might contribute to the development of cancer and other age-related pathologies [246]. Interestingly, in aged rats, hypomethylation of histones and DNA occurs in the primary (thymus) organ involved in immune response, and histone hypomethylation also occurs in the secondary (spleen) organ involved in immune response [246].

As mentioned in the beginning of this review, SIRT1 is a HDAC that is involved in circadian rhythm generation and memory. While a variety of epigenetic changes occur during aging, SIRT1 activity is a key player in the aging-associated phenotype. For example, SIRT1 is neuroprotective against neurodegenerative age-associated pathologies such as AD; thus, it has been implicated in promoting longevity and healthy aging [102, 247, 248]. Specifically, SIRT1 activation decreased neurodegeneration, the amount of beta amyloid formed, and improved memory in animal models of AD [249, 250]. Furthermore, SIRT1 expression is attenuated in senescent cells, the thymus, the testis, and in the skin of a transgenic mouse model that accelerates aging [239, 242, 251]. Conversely, mice that overexpress SIRT1 in the brain live longer and display delayed aging compared to wild-type animals [252].

#### The epigenome in the SCN and hippocampus is affected by aging

In aged rodents, epigenetic changes occur in areas of the brain that modulate memory and circadian rhythms. As highlighted in the beginning of this review, SIRT1 and DNA methylation are both necessary for circadian rhythms and memory. Aging elicits similar changes to these epigenetic mechanisms in the hippocampus and SCN. Although this is a burgeoning research field, convincing evidence from several studies indicates that the epigenome in these brain areas is affected by aging.

#### Memory

Recent evidence suggests that the epigenetic changes that underlie learning and memory are affected by aging. For example, shortly after spatial learning and contextual fear conditioning, histone 4 acetylation in the hippocampus of aged mice (CA1 and DG) is down-regulated and HDAC inhibitors rescue acetylation and the concomitant learning impairments [130, 195]. Age-related dysregulation of learning-induced histone acetylation is specific to the brain area modulating that specific learning task. For example, the age-related acetylation dysregulation is only seen in the hippocampus or striatum when spatial (hippocampal-dependent) and cue-based (striatal-dependent) versions of the MWT are used, respectively [130]. When mice have the option of using a place or cue strategy in MWT, a histone deacteylase inhibitor delivered to the CA1 region of the hippocampus results in a bias for a hippocampal-dependent place strategy, but this effect is lost in aged mice [253].

Similarly, Penner and colleagues [196] observed altered hippocampal DNA methylation of an immediate early gene required for memory consolidation, the activity-regulated cytoplasmic gene (ARC), which likely contributes to reduced *ARC* mRNA expression and concomitant impairments in the MWT. SIRT1 protein levels in the hippocampus are also decreased in aged rats, while expression in the cerebral cortex is unaffected [254]. This attenuated hippocampal expression is due to post-translational modifications, as SIRT1 mRNA expression is maintained in aged rats [254]. Decreased SIRT1 in the hippocampus might contribute to age-related memory impairments, as transgenic mice with SIRT1 deletions are impaired in hippocampal-dependent tasks [104, 105]. However, until mice with a SIRT1 deletion specific to the hippocampus are trained in a hippocampal-dependent task, this claim cannot be verified.

#### Circadian rhythms

Recent studies have indicated that epigenetics are involved in the circadian rhythm dysfunction that occurs with aging. Readers are encouraged to see the review by Orozco-Solois and Sassone-Corsi [13], which discusses how the involvement of epigenetics in circadian rhythms might affect aging. In the SCN of aged mice, the reduction of a primary DNA methyltransferase was associated with disrupted circadian rhythms and the inability to entrain to a slightly shorter light-dark cycle [91]. As changes in DNA methylation regulate circadian rhythm entrainment, the authors hypothesized that age-related changes in SCN DNA methylation might explain why chronotype changes with age [91, 110, 113, 114].

In addition to age-associated cognitive decline, Chang and Guarente [109] unequivocally demonstrated that SIRT1 degradation contributes to circadian dysfunction in aged mice. As the deletion of SIRT1 expression in the brain creates a free-running period and a disrupted activity pattern that is similar to that in aged mice, not surprisingly, the authors observed decreased SIRT1, BMAL1, and PER2 expression in the SCN of aged mice. The authors also observed a partial rescue in aged mice's ability to entrain to a photoperiod shift when SIRT1 was overexpressed in the brain [109].

#### Might age-induced epigenetic changes in circadian rhythms contribute to memory impairments?

**Figure 1:**
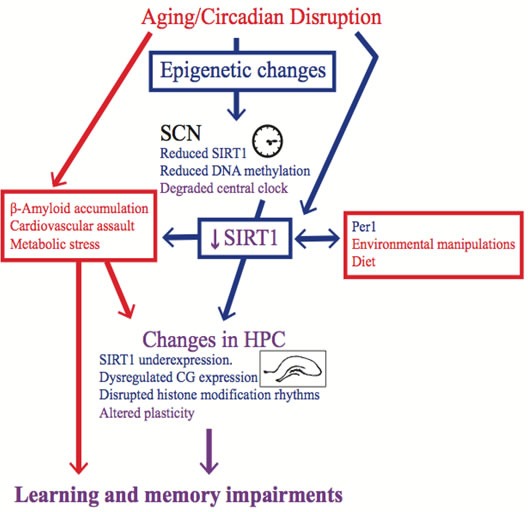
Epigenetic changes are a possible mechanism for the memory impairment induced by circadian rhythm disruption The blue items are either epigenetic mechanisms or affected by changes in the epigenome, whereas the red items either elicit non-epigenetic mechanisms or are affected by them. The purple items are believed to be effected by both epigenetic and non-epigenetic mechanisms. Factors that induce circadian rhythm disruption, such as aging or shift work, change the epigenome in the SCN, and these changes contribute to SCN dysfunction. SCN dysfunction then affects the epigenome in peripheral oscillators, such as the hippocampus, which contribute to learning and memory impairments. Other factors, such as diet and environmental manipulations could also create learning and memory impairments by changing the epigenome in the hippocampus. It should be noted however that some of these environmental manipulations might also influence circadian rhythms. Alternatively, in addition to circadian rhythm disruption, the memory impairment induced by aging could be a result of other risk factors that impact hippocampal functioning. However, these risk factors are likely exacerbated by circadian rhythm disruption, and this effect could be mediated by changes in the epigenome.

While there are age-induced changes in some epigenetic mechanisms in the SCN and hippocampus, it is unclear if these changes are contingent on each other. Based on the behavioural data from our lab, which suggests that the degree of circadian rhythm entrainment influences memory in both young and aged rodents, we believe that the SCN becomes dysfunctional with age and this circadian rhythm disruption then affects hippocampal functioning, which elicits cognitive impairments. There are a myriad of ways in which circadian rhythm dysfunction could affect hippocampal functioning [2, 58]; we discuss one possibility in which epigenetic changes in the SCN elicit circadian dysfunction, which then induces epigenetic changes in the hippocampus (see Figure 1).

As mentioned above, DNA methyltransferase and SIRT1 expression are attenuated by aging in the SCN, and these mechanisms each affect the ability of mice to entrain to a new photoperiod [91, 109]. Therefore, SCN epigenetic changes in aged animals can elicit circadian rhythm dysfunction, and there is recent evidence that suggests that circadian rhythm dysfunction can affect the hippocampal epigenome. For example, in the hippocampus, *Per1* is required for the rhythmic oscillations of various histone modifications, such as SIRT1, and learning-induced epigenetic changes [32]. These data suggest that clock genes modulate epigenetic changes in the hippocampus. These results should be interpreted with caution, as the *Per1* mutation was global, so it is unclear if the deficits were due to disrupted oscillations in the hippocampus or were caused by aberrant outputs from the SCN.

Environmental manipulations that elicit circadian rhythm disruption might help determine if hippocampal epigenetic changes induced by circadian disruption are due to the uncoupling of the hippocampus with the SCN (see Figure 1). As previously mentioned, Azzi and colleagues [91] demonstrated that DNA methylation fluctuates in the SCN depending on the photoperiod and regulates entrainment to a new photoperiod. However, SIRT1 expression has not been evaluated in the SCN or hippocampus of rodents in which circadian rhythms have been disrupted by altering the light cycle. Nonetheless, in addition to the transgenic mouse model mentioned above, several studies have used manipulations that likely have induced circadian disruption. A relatively brief period of sleep deprivation alters the phase of locomotor activity rhythms [255] and large reductions in the amplitude of electrical activity in the SCN that lasts for hours after sleep deprivation [256]. With this in mind, five days of total sleep deprivation resulted in attenuated hippocampal SIRT1 expression in rats with concomitant impairments in a rapid acquisition version of the MWT [257]. Although these authors did not measure circadian rhythm entrainment, it is inconceivable that these rats were not experiencing circadian rhythm dysfunction. Similarly, high-fat diets alter the period of the locomotor activity rhythm and disrupt clock gene oscillations in some peripheral tissues [258]. Mice given a high-fat diet for 23 weeks had reduced SIRT1 expression in the hippocampus and concomitant impairments in the object location memory task, which is thought to be a hippocampal-dependent task [259]. Again, the authors did not measure circadian entrainment, but these animals most likely had disrupted circadian rhythms. These data suggest that circadian rhythm disruption can cause epigenetic dysfunction in the hippocampus, which would then affect the signal transduction pathway that is required for memory acquisition, consolidation, and retrieval.

There is another way in which epigenetic-induced SCN dysfunction could contribute to age-related cognitive impairment. We have proposed that age-related dementia or cognitive decline is due to the combined effect of risk factors that affect hippocampal functioning [260–262]. The idea is that cognitive impairments and hippocampal pathology are exacerbated when these risk factors are presented concomitantly instead of in isolation [260–262]. Circadian rhythm disruption could make the hippocampus more vulnerable to damaging factors [260–262]. Although preliminary, we recently demonstrated that mini-hippocampal strokes elicited more degenerating neurons and smaller hippocampal volumes when they were given to rats that had circadian rhythm disruption compared to rats with entrained rhythms (Gidyk et al., under review). Mechanistically, decreased SIRT1 in the hippocampus elicited by circadian rhythm disruption and/or aging could make the brain more susceptible to damaging factors (see Figure 1). For example, as previously mentioned, SIRT1 is a neuroprotective agent that inhibits beta-amyloid formation [102, 248–250]. Whether circadian rhythm dysfunction induced by epigenetic changes is disrupting memory in aged individuals via epigenetic changes in the hippocampus or by increasing hippocampal vulnerability, this mechanism deserves further investigation.

#### Circadian rhythms and memory are rescued by manipulations that affect the epigenome

The notion that drugs and environmental manipulations affect the epigenome is particularly enticing. Caloric restriction in aged animals increases longevity, improves memory/cognition, and reduces markers and the onset of neurodegenerative disease [102, 239]. Age-related impairments in hippocampal-dependent memory, LTP, and NMDA receptor expression in the hippocampus also do not occur in old rats that have received life-long caloric restriction [263, 264]. Caloric restriction also can increase or decrease SIRT1 expression depending on the brain region [265, 266]. Relatively brief periods of calorie restriction are sufficient to increase SIRT1 protein levels in the hippocampus [254] of aged rats or in the cortex and hippocampus of adult mice [265]. In addition to boosting SIRT1 expression, calorie restriction also improves insulin function and affects the mechanistic target of rapamycin (MTOR) pathway [267, 268]. The nutrient sensing MTOR pathway affects cell growth/division and while activation of this pathway is initially positive, during aging MTOR activity is deleterious because it elicits cellular aging [102, 268]. Calorie restriction promotes healthy aging by inhibiting the MTOR pathway and SIRT1 is necessary for this effect [102, 268].

Similar to food restriction, calorie restriction can also synchronize peripheral oscillators; however, unlike food restriction, calorie restriction can entrain the SCN [269–272]. Interestingly, calorie restriction also increases SIRT1 expression it the SCN and hippocampi of adult mice [265, 266]. Froy and Miskin [272] discuss how improved circadian rhythmicity likely contributes to increased longevity induced by calorie restriction. The finding that calorie restriction affects circadian rhythms in aged rats, mice, and rhesus monkeys supports this notion [273–275]. Furthermore, in contrast to wild-type mice, the period of circadian rhythms and diurnal pattern of food intake is maintained during aging in αMUA mice, which consume 20% to 30% less food than wild-type mice [142, 276]. With this in mind, it would be interesting to assess circadian rhythms and memory in adult calorie-restricted rats exposed to a photoperiod-shifting paradigm.

Melatonin also has an epigenetic effect that influences both circadian rhythms and memory. Melatonin is attenuated in aged female rats and humans [151, 153] and regulates circadian rhythmicity; recently, it has been suggested that melatonin can influence memory [102, 257]. Exogenous melatonin reduces tumor growth that is elicited by circadian rhythm disruption and also mitigates the accompanying decrease in global DNA methylation [126]. In addition to reducing tumor growth, normal melatonin rhythmicity or exogenous melatonin given to rats with disrupted melatonin rhythms prevents resistance to the breast cancer drug tamoxifen [277]. Melatonin has been linked to the SIRT1 pathway and is thought to epigenetically silence genes [73, 102, 257]. Jenwitheesuk and colleagues [102] suggest that the memory impairment that occurs in aged individuals might be due to diminished circadian expression of melatonin and the resultant effect on hippocampal SIRT1 expression. The finding that melatonin partially rescues SCN clock gene expression and hippocampal SIRT1 expression in sleep-deprived rats supports this hypothesis [171, 257].

## CONCLUSIONS

Circadian rhythms influence memory in young and old subjects. Epigenetic mechanisms are necessary for both circadian rhythms and memory, and these mechanisms are affected by aging. It is possible that circadian rhythm disruption, partially induced by age-associated changes in the epigenome, affects epigenetic mechanisms in the hippocampus, which results in memory impairments. Alternatively, circadian rhythm disruption might precipitate other pathologies that affect hippocampal function. If SCN lesions and environmental light manipulations disrupt hippocampal epigenetic mechanisms, then the hypothesis that hippocampal epigenetic mechanisms are disrupted by circadian rhythm degradation would be strengthened. SCN and hippocampal tissue specific knockouts of clock genes and molecules involved in epigenetic function would also help answer this question. Regardless, with this question, it is difficult to infer causation, as aging affects many processes that might impact hippocampal epigenetic function. It would be interesting to see if the degree of circadian rhythm entrainment in aged animals correlates with hippocampal epigenetic function. Nonetheless, epigenetics as a possible mechanism for how circadian rhythm disruption can affect hippocampal-dependent memory is an intriguing hypothesis that deserves further investigation.
